# Effect of okra bast fillers on biodegradation properties of poly(vinyl alcohol) composites

**DOI:** 10.55730/1300-0527.3512

**Published:** 2022-09-07

**Authors:** Gazi Md Arifuzzaman KHAN, Nazire Deniz YILMAZ

**Affiliations:** 1 Department of Applied Chemistry and Chemical Engineering, Islamic University, Kushtia, Bangladesh; 2 Department of Textile Engineering, Faculty of Engineering, Uşak University, Uşak, Turkey

**Keywords:** Agroresidue, biocomposite, biodegradation, mechanical properties, okra bast fiber, poly(vinyl alcohol)

## Abstract

This paper focuses on the effect of okra bast fillers on biodegradation of poly(vinyl alcohol) composites. Fibers were obtained from okra plant stems via biological degumming and subjected to different chemical treatments such as scouring, alkalizing, maleic anhydride treatment, and vinyl acetate grafting. The fibers were ground and PVA-okra bast filler composites were produced at 20 wt% fiber load via the solution casting method. The obtained composites were tested in terms of mechanical properties and exposed to biodegradation in soil. Effects of okra bast filler addition and chemical treatments on changes in mass, breaking strength, elasticity modulus, and breaking elongation of composites upon soil biodegradation were determined. The composites can be considered for utilization in packaging and agricultural applications.

## 1. Introduction

Intensive attention has been given to biodegradable materials in a number of different areas based on increased environmental concerns and tightening regulations [1]. Biodegradable materials can be obtained from forests (wood, lumber, etc.), agriculture practices (agricultural byproducts, crop surpluses, agricultural wastes), or can be chemically synthesized from biobased sources (like poly lactic acid) and even from petroleum (polycaprolactone; polybutylene succinate) [2,3].

Biodegradable materials are generally prepared in the form of composites form in order to acquire improved performance characteristics. Polymeric matrices can be reinforced with natural fibers and/or fillers to achieve better tensile properties, biodegradation behavior and cost-effectiveness. The packaging industry and agriculture are two areas where biodegradable materials and biocomposites show promise. Some specific uses for packaging can be given as single-use cups and containers; whereas some agricultural uses include mulch films and greenhouse components [3].

Poly(vinyl alcohol), which has been first developed in 1924 by Herman and Haehnel [4], is a biodegradable, biocompatible, bioinert and water soluble [5], thermoplastic polymer. It is generally prepared from hydrolysis of polyvinyl acetate [6] which can be produced via different routes based on bio or petro-based raw materials [https://www.helm.com.tr/tr/is-kollari/kimyasallar/ueruenler/ayrintilar/HTR-Vinyl-Acetate-Monomer?ai%5Bd_pos%5D= (accessed Mar. 24, 2022).]. The molecular formula of PVA is (C_2_H_4_O)_n_ [7]. It shows high strength, ductility, and flexibility, while the physical and chemical characteristics show changes based on the degree of polymerization and hydrolysis, and grade. The outstanding characteristics which render PVA an ideal alternative to nonbiodegradable polymers can be listed as good chemical resistance, optical properties (transparency/translucency), film forming ability, and biocompatibility [4]. It has found use in packaging, wood, paper, paint, textiles industries [8], and demanding applications in the biomedical field including surgical threads, contact lenses, wound dressing, and drug delivery [6].

Due to its polarity, PVA shows promise for use with plant fibers which also exhibit polar chemical structure [3]. Plant fibers, based on renewable sources and presenting biodegradability, are viable environmentally-friendly alternatives to their synthetic counterparts [9]. Accordingly, research studies are ongoing focusing on the utilization of PVA with lignocellulosic fibers.

Cinelli et al. [10] worked on PVA-based composites with agroresidual corn fibers and corn starch. They studied mechanical properties, and change in mechanical properties upon 1 h submerging in water, and one year at 23 °C at 50% relative humidity. Imam et al. [3] studied the chemical, thermal, aqua permeability, and biodegradation properties of PVA biocomposites incorporating cornstarch and orange fibers. Kalambettu et al. [11] produced composites of PVA with palm olive leaf fiber and investigated their mechanical, water absorption as well as soil- and plate-biodegradation characteristics.

Ching et al. [1] investigated mechanical properties of oil palm empty fruit bunch fiber-reinforced PVA biocomposites and effects of thermal treatments on their tensile performance.

Ali et al. [12]produced PVA biocomposites reinforced with kenaf fibers. They studied tensile and flexural properties of the composites and effects of chemical treatments. Parvin et al. [13] investigated tensile properties of PVA composites reinforced with fibrillated cellulose fiber.

Other than a handful of plant fibers, no other research effort devoted to the use of natural fibers in PVA-based composites has been found by the authors. Agroresidual fibers constitute a special position among other fibers, as they are obtained as a byproduct of agriculture practice allocated mostly for food production. Okra bast fibers obtained from stems of okra plant exhibit properties close to textile bast fibers such as flax and jute [14–16]. In the current study, fibers extracted from okra stem via biological degumming were scoured. Chemical modification processes, namely, alkalization, maleic anhydride and vinyl acetate treatments were applied. Obtained fibers were used as fillers in PVA composites. Composites were subjected to biodegradation and its effects on mass and tensile properties have been investigated.

## 2. Materials and methods

### 2.1. Materials

Okra stems were obtained from a farm in Denizli providence, Turkey. Maleic anhydride and vinyl acetate monomers were supplied from Sigma-Aldrich. Polyvinyl alcohol 72,000 powder (98% hydrolyzed) was purchased from Merck, Germany. Solvents and reagents utilized in the current study were analytical grades.

### 2.2. Methods

**Figure 1:**
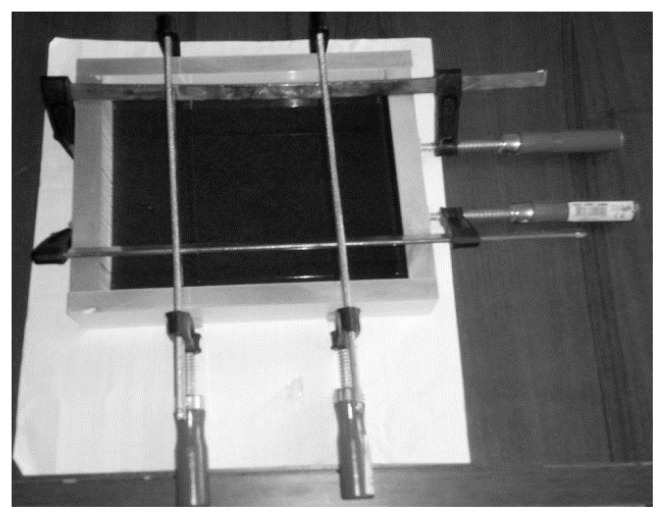
A mold used for biocomposite preparation by solution casting method.

**Figure 2:**
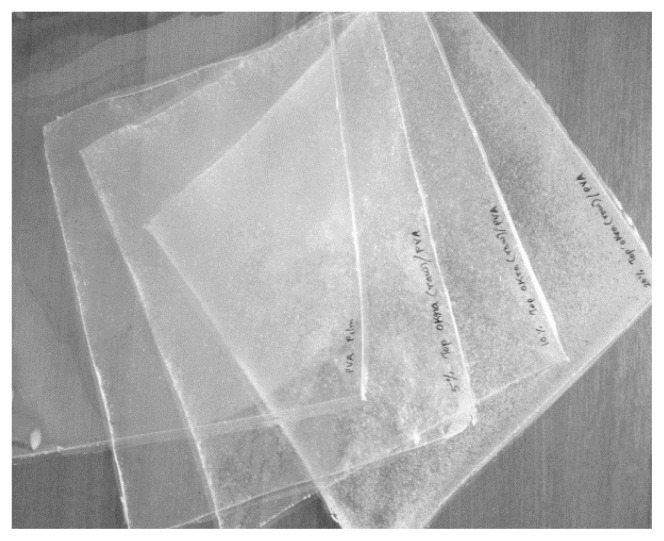
Prepared okra fiber reinforced PVA composite samples.

The collected okra stems are evenly divided in three sections: upper (U), middle (M), and lower (L) sections. Okra fibers were extracted separately for these three sections in different containers. Okra bast fiber extraction and chemical treatments have been conducted as explained in [17]. Okra bast fibers were first chopped and then smashed in a ceramic mortar. PVA solution was obtained by dissolving PVA powder in distilled water via continuous stirring with a magnetic stirrer for a duration of 1–1.5 h at 80 °C. The liquor ratio was 1:20 PVA/aqua. Different samples of okra fibers based on location on the stem and chemical pretreatments at 20wt% (with respect to PVA) were included to the PVA solution as shown in Table and stirring was continued for another 30 min at 80 °C. The obtained mixtures were cast on a mold as shown in Figure 1. The mixtures were left to dry where the solvent (aqua) fully evaporated at room temperature for 1–2 days. The obtained composite films shown in Figure 2 were then released from the mold. As a control sample, an aqueous solution of PVA without the addition of fibers was cast on mold. Prior to characterization, biocomposites were measured in terms of dimensions and kept in an oven at 80 °C for 2.5 h.

The tensile testing of samples was performed according to ASTM D 638. Sample dimensions were 100 mm × 10 mm. A Tinius Olsen H10KT^(R)^ Tester, US with QMat for Textiles^(R)^ software, were used with an initial grip separation of 50 mm, and the loadcell was 500 N. Seven replications for each sample were conducted.

Soil degradation was carried out by burying composite samples at 100 mm × 10 mm dimensions in a plastic soil container (44 × 30 × 23 cm^3^) filled with agricultural soil at pH 7. Relative humidity was maintained at 90%–95% by sprinkling water at room temperature each day. Soil degradation duration was 14 days. After completion, the samples were taken out of the soil and then washed and left to dry in ambient air for 3 days. After then, they were kept in an oven at 80 °C for 2.5 h. Biodegradation was evaluated by measuring loss in mass and tensile properties of the composites. Three replications were conducted for tensile testing of biodegraded specimens.

Scanning electron microscopy (SEM) images of okra bast fibers and fractured okra fiber-PVA composites were taken by using a Zeiss EVO LS 10 SEM microscope, at 20 kV and 300 ×, 500 ×, 1000 × and 5000 × magnifications. Samples were coated with gold 5-nm thick by using a Cressington Auto 108 model sputter coater.

## 3. Results and discussion

Okra bast fibers-reinforced PVA composites have been investigated in terms of morphology, tensile properties, and biodegradation behavior.

### 3.1. Morphology

**Figure 3:**
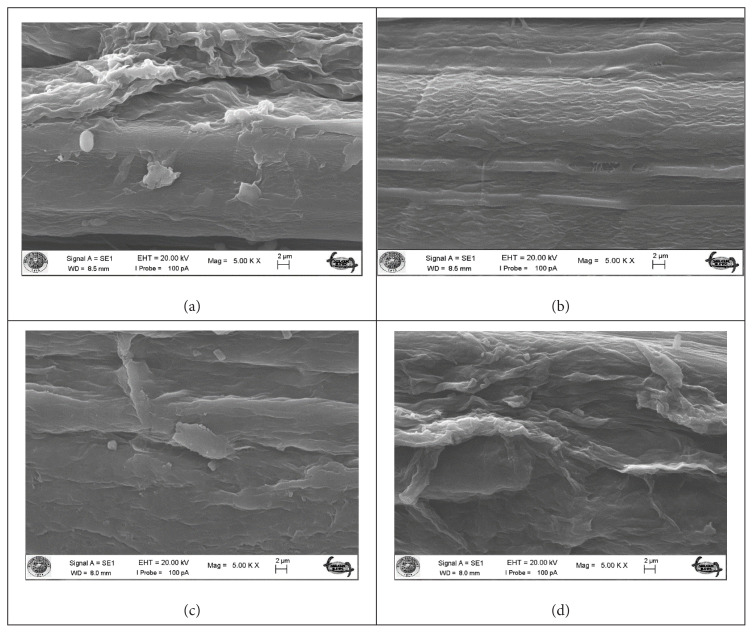
SEM images of okra bast fibers longitudinal at 5000 × magnification (a) scoured okra bast fiber, (b) alkalized okra bast fiber, (c) maleic anhydride treated okra bast fiber, and (d) vinyl acetate grafted okra bast fiber.

**Figure 4:**
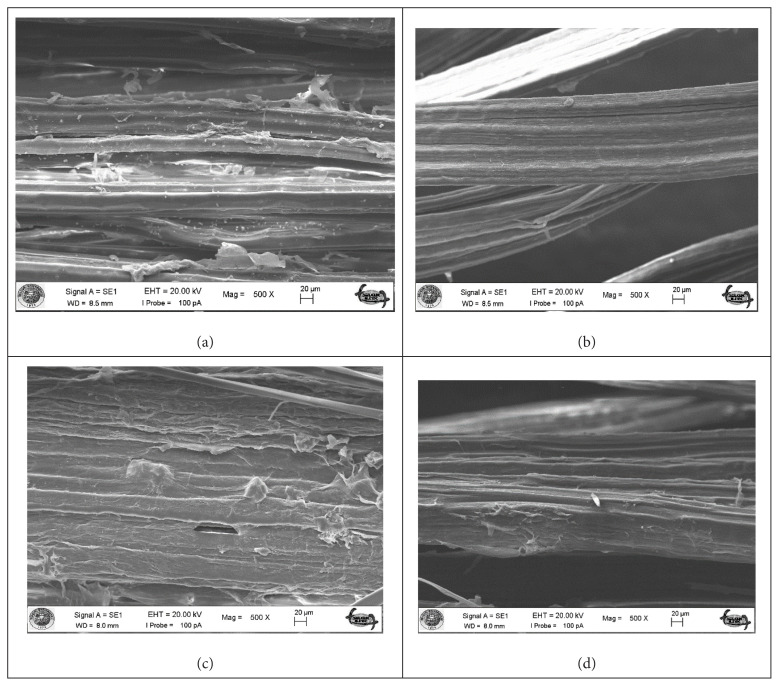
SEM images of okra bast fibers longitudinal at 500 × magnification (a) scoured okra bast fiber, (b) alkalized okra bast fiber, (c) maleic anhydride treated okra bast fiber, and (d) vinyl acetate grafted okra bast fiber.

**Figure 5:**
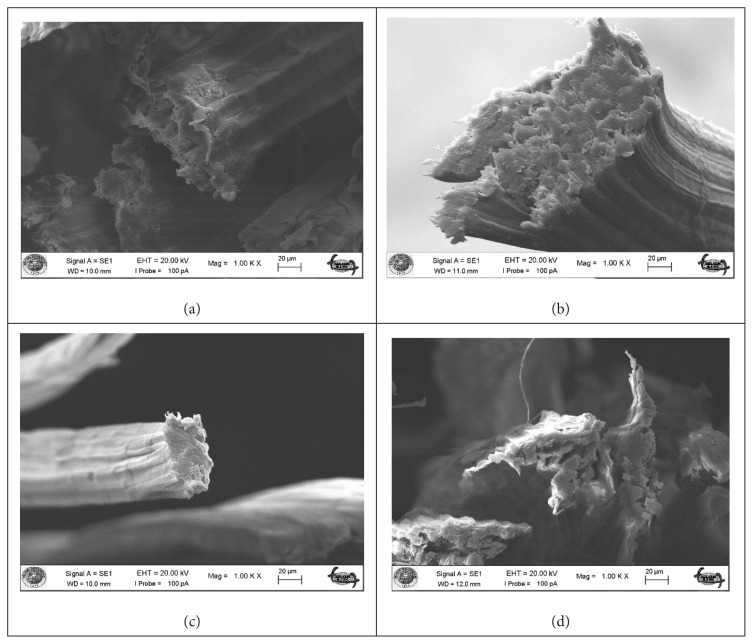
SEM images of okra bast fibers cross-section at 1000 × magnification (a) scoured okra bast fiber, (b) alkalized okra bast fiber, (c) maleic anhydride treated okra bast fiber, and (d) vinyl acetate grafted okra bast fiber.

**Figure 6:**
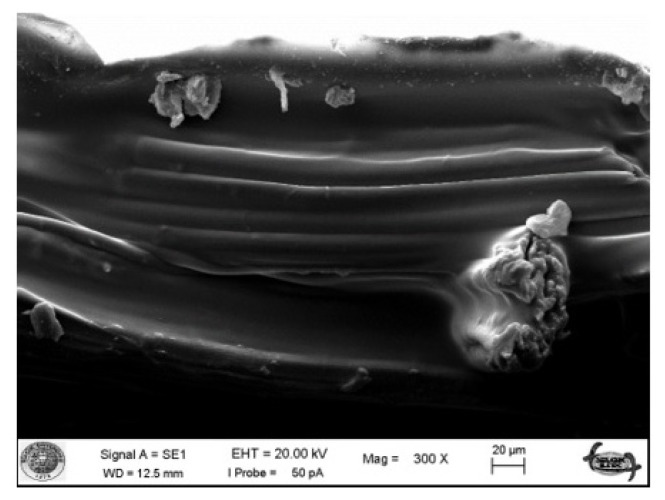
SEM image of BD20U okra bast fiber-PVA fracture surface at 300 × magnification.

The morphological structure of okra bast fibers is shown in SEM images (Figures 3–5). Figure 3 shows the fibers in longitudinal orientation at 5000 × magnification to present a closer look at surface topography, whereas Figure 4 exhibits longitudinal wholistic observation at 500 × magnification. Figure 5 shows cross-sectional images of okra bast fibers. The figures reflect the complex multifibrillar structure of okra bast fibers including fibrils and extracellulosic binding substances. The fibrils show orientations parallel to the axis of the technical fiber and to each other. Scoured okra bast fiber surface presents higher amount of surface impurities in comparison to alkalized fiber. This hints the effect of alkalization in removing extracellulosic components from the fiber. Duman et al. [18] also reported decreased okra fiber weight and coarseness upon alkalization, which is in agreement with the current study. From Figure 4, and more closely from Figure 3, an extra layer is observed for maleic anhydride and vinyl acetate treated fibers in comparison to alkalized fibers. The treatments may tend to fibrillation of fibers; however, this cannot be proved from SEM images, which can cover a very limited number of fibers showing very high inherent variability. The cross-sectional shape of okra fibers resembles an irregular ellipse with varying area depending on the number of fibrils contained within. The high variability of okra fibers can be observed from the presented SEM images. Figure 6 depicts fracture surface of 20 wt% scoured okra fiber reinforced PVA composite. The image mainly includes matrix-rich region. This hints agglomeration and uneven dispersion of okra fillers in the PVA matrix.

### 3.2. Tensile properties of composites

**Figure 7:**
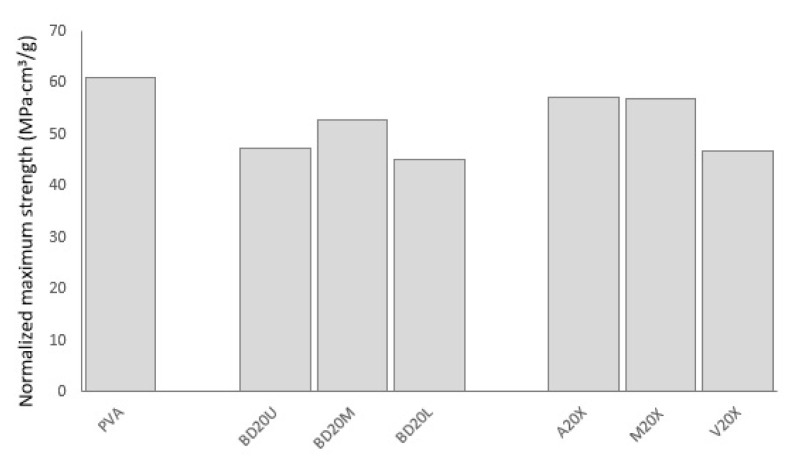
The normalized maximum strength of PVA composites in comparison to neat PVA.

Figure 7 shows maximum strength of PVA composites in comparison to neat PVA normalized by density. None of the composites results in maximum strength higher than the 100% PVA control. In essence, 20wt% is a high fiber loading rate. Lower loading rates may result in better tensile performance. Among chemically treated fibers, alkalized and maleic anhydride treated fibers give better results compared to untreated and vinyl acetate treated fibers. Better results obtained from alkalized fibers may be due to the effect of alkalization on removing surface impurities, in agreement with the morphology analysis shown with the SEM images. The surface impurities may have hindered effective bonding between the fibers and the matrix. Among untreated fibers, okra bast fibers obtained from the middle section of the okra stem result in higher strength, than those from the upper or lower parts.

**Figure 8:**
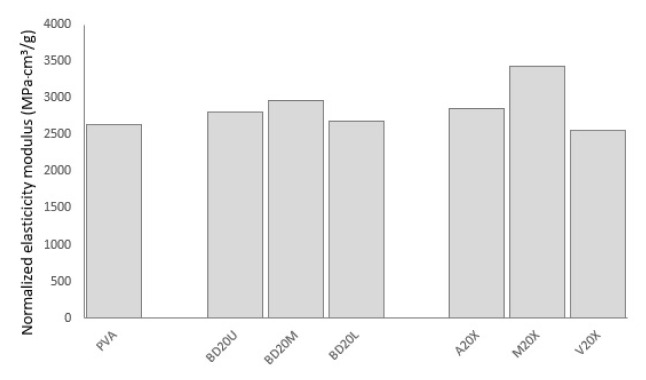
Normalized elasticity modulus of PVA composites in comparison to neat PVA.

Normalized modulus of elasticity presents a similar trend as can be seen in Figure 8; however, this time 20 wt% fiber load results in higher performance in comparison to neat PVA other than vinyl acetate-grafting. Kalambettu et al. [11] also reported increased stiffness of PVA with incorporation of lignocellulosic fibers. Maleic anhydride treatment gives the highest normalized elasticity modulus, surpassing that of alkalization. This fact may be due to the fact that unstrained alkalization may lead to higher crimp and helical orientation of fibers which may have led to decrease in elasticity modulus [17], as well as better interfacial bonding for maleic anhydride treated fibers compared to alkalized ones.

### 3.3. Biodegradation

Biodegradation was defined by Mollasalehi [4] as “a decomposition process through the action of microorganisms, which results in the recycling of the polymer carbon, followed by mineralization (that is the production of CO_2_, H_2_O, and salts) of compounds and the creation of new biomass”. Bio-based- as well as synthetic polymers can biodegrade based on the activity of bacteria (such as *Pseudomonas putida* VM15A, *Pseudomonas sp.* VM15C) [8] and fungi (including *Phanerochaete chrysoporium* and *Pycnoporus cinnabarinus*) in water [7], landfill, compost, and soil environments under aerobic as well as anaerobic conditions [4]. Polymer properties such as the degree of polymerization [19], microorganism type, temperature, pH, O_2_ and CO_2_ content are among the factors affecting biodegradation [4,11].

PVA biodegrades in a stepwise fashion when subjected to extracellular and intracellular enzymes (such as PVA-oxidase and PVA-dehydrogenase) of PVA-degrading microorganisms. The first-step products of PVA degrading are ketones, alcohols and fatty acids generated as a result of C-C bond breaking effect of extracellular enzymes, which in turn degrade into acetic acid, CO_2_ and hydrogen that can finally be consumed by methanogens. Then, PVA is completely biodegraded [4,7,8].

In comparison to physical and chemical degradation including incineration [8], biodegradation exhibits better economy, and less complicated procedures. Furthermore, it does not emit additional pollutants [7]. On the other hand, a disadvantage of biodegradation is unstable biodegrading microorganism effectiveness [7].

**Figure 9:**
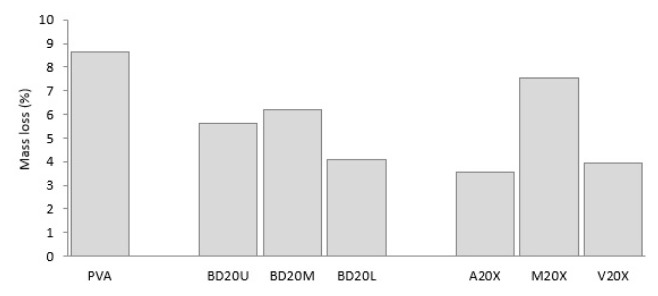
Mass loss PVA composites upon soil degradation in comparison to neat PVA.

**Figure 10:**
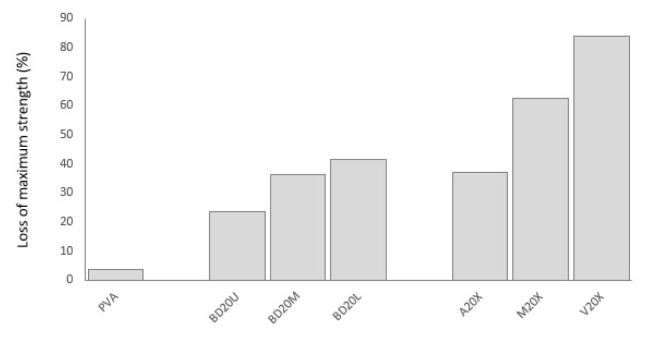
Maximum strength loss PVA composites upon soil degradation in comparison to neat PVA.

**Figure 11:**
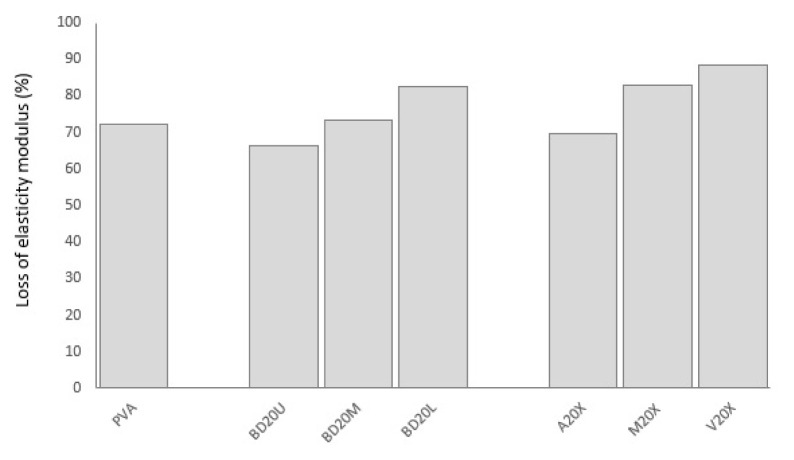
Elasticity modulus loss PVA composites upon soil degradation in comparison to neat PVA.

**Figure 12:**
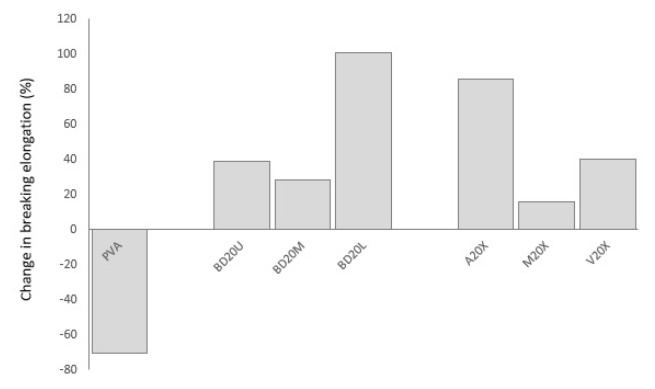
Change in breaking elongation of PVA composites upon soil degradation in comparison to neat PVA.

As seen from Figures 9–12, okra bast fiber addition acted in a peculiar manner on the biodegradation behavior of PVA composites. Figure 9 shows that fiber addition substantially decreases mass loss; in other words, okra fiber-reinforced composites retained more of their initial mass in comparison to that of neat PVA. Cellulose backbones of okra bast fibers may have formed chains that maintained integrity of the structure. Kalambettu et al. [11] also argued that lignocellulosic fillers hold PVA matrix together acting as a binder. Minimum mass loss was recorded for alkalized okra fiber-reinforced PVA. As seen from SEM analyses, alkalization led to the elimination of extracellulosic substances from the surface of the fibers. Along with enhancing fiber-matrix bonding, this may also lead to the elimination of biological materials which may act as a nutrition source for PVA-degrading microorganisms.

Biodegradation is reflected in tensile properties deterioration in a substantially different manner. The addition of okra bast fibers led to increased losses in tensile indicators. This is especially amplified for maximum strength loss. Okra fibers may have acted as carbon element supply, which is a nutrient element necessary for biodegrading bacteria [7]. Another reason may be that the biodegradation rate of ground okra fibers may be faster than biodegradation of PVA, which is slow compared to other biodegradable polymers [4]. In the current study, whereas neat PVA gave a 3.6% strength loss, composites present losses in the range of 23%–83%. There was much lower difference between neat PVA and fiber-reinforced PVA composites when it comes to elasticity modulus: 72% vs. 66%–89%, respectively. Imam et al. [3], Kalambettu [11] and Chiellini et al. [20] also reported accelerated biodegradation of PVA composites upon reinforcing with lignocellulosic fibers such as palm olive leaf fiber, sugar cane bagasse and orange fibers. In the current study, it is seen that strength and modulus loss is in ascending order for okra bast fibers obtained from upper<middle<lower sections of the okra plant. Fibers obtained from the lower parts may include a higher amount of extracellulosic substances which accelerate biodegradation compared to those obtained from the upper parts, presenting more nutrients to degrader microorganisms and hindering effective bonding with PVA. Change in elongation break happened in a different fashion. Whereas PVA lost 70% of its elongation at break rate, fiber-reinforced composites exhibit 28%–101% increase in elongation. PVA, which exhibits ductile nature, faced elongation loss; on the other hand, okra bast fibers, which are inherently rigid and brittle, gained ductility upon soil degradation. Former literature on biodegradation of plant fiber-reinforced PVA also presented complicated effects. Imam et al. [3] reported a negative effect of fiber addition on mass loss of PVA composites upon biodegradation similar to the current study together accelerated biodegradation. On the other hand, Tan et al. [21] found increased CO_2_ production for fiber added PVA composites reflecting increased biodegradation in soil.

## 4. Conclusion

Effects of okra bast fillers on biodegradation of poly(vinyl alcohol) (PVA) composites have been investigated. The presence of okra fillers resulted in decreased mass loss, but increased tensile strength reduction in biodegraded PVA samples. The effect of okra fillers can be interpreted as a backbone that maintains the integrity of the material together with acting as a nutrient supply to microorganisms accelerating their biodegrading activity. Neat PVA, originally exhibiting ductility, loses elongation rate upon biodegradation. On the other hand, PVA reinforced with okra fillers, a rigid reinforcing material, gains elongation rate at break after biodegradation. Among biodegraded composites of PVA reinforced with different treated okra fillers, alkalized okra fillers exhibit minimum mass loss, strength and rigidity, emphasizing the effect of the alkalization process in eliminating extracellulosic surface substances, that are nutrients for biodegrading bacteria as well as hindering effective interfacial bonding with PVA. Untreated okra bast fillers obtained from the upper sections of okra stem led to the lowest reductions in tensile strength and elasticity modulus compared to that of PVA composites reinforced with okra fillers extracted from the middle and lower parts of okra stem. The composites can be considered for utilization in packaging and agricultural applications, where biodegradation can be manipulated by addition of okra fillers from different parts of okra plant stem and subjected to different chemical pretreatments.

## Figures and Tables

**Table:** Production parameters of okra bast fiber reinforced composites.

Sample code	Location in okra plant	Fiber extraction	Scouring	1^st^ chemical treatment	2^nd^ chemical treatment	Fiber loading wt (%)	PVA content wt (%)
PVA	N/A	N/A	N/A	-	-	0	100
BD20U	Upper	Biological degumming	Na_2_CO_3_	-	-	20	80
BD20M	Middle	Biological degumming	Na_2_CO_3_	-	-	20	80
BD20L	Lower	Biological degumming	Na_2_CO_3_	-	-	20	80
A20X	Mixed	Biological degumming	Na_2_CO_3_	Alkalization	-	20	80
M20X	Mixed	Biological degumming	Na_2_CO_3_	Bleaching	Maleic anhydrite	20	80
V20X	Mixed	Biological degumming	Na_2_CO_3_	Bleaching	Vinyl acetate	20	80
